# Bacterial amidohydrolases and modified 5-fluorocytidine compounds: Novel enzyme-prodrug pairs

**DOI:** 10.1371/journal.pone.0294696

**Published:** 2023-11-30

**Authors:** Viktorija Preitakaitė, Povilas Barasa, Agota Aučynaitė, Gediminas Plakys, Martyna Koplūnaitė, Simona Zubavičiūtė, Rolandas Meškys

**Affiliations:** 1 Department of Molecular Microbiology and Biotechnology, Institute of Biochemistry, Life Sciences Center, Vilnius University, Vilnius, Lithuania; 2 Department of Biological Models, Institute of Biochemistry, Life Sciences Center, Vilnius University, Vilnius, Lithuania; New York University Langone Health, UNITED STATES

## Abstract

Gene-directed enzyme prodrug therapy is an emerging strategy for cancer treatment based on the delivery of a gene that encodes an enzyme that is able to convert a prodrug into a potent cytotoxin exclusively in target cancer cells. However, it is limited by the lack of suitable enzyme variants and a scarce choice of chemical bonds that could be activated. Therefore, this study is aimed to determine the capability of bacterial amidohydrolases YqfB and D8_RL to activate novel prodrugs and the effect such system has on the viability of eukaryotic cancer cells. We have established cancer cell lines that stably express the bacterial amidohydrolase genes and selected several *N*^4^-acylated cytidine derivatives as potential prodrugs. A significant decrease in the viability of HCT116 human colon cancer cell lines expressing either the YqfB or the D8_RL was observed after exposure to the novel prodrugs. The data we acquired suggests that bacterial YqfB and D8_RL amidohydrolases, together with the modified cytidine-based prodrugs, may serve as a promising enzyme-prodrug system for gene-directed enzyme prodrug therapy.

## Introduction

Amidohydrolases are a large class of enzymes that catalyze the hydrolysis of various linear and cyclic amides [1]. These enzymes are widespread in animals, plants, and microorganisms, where they are involved in several metabolic pathways, most commonly in nucleotide metabolism [2]. Due to their high stereo- and regioselectivity, amidohydrolases have a wide range of bio-applications not only in chemical synthesis, but also in the food and cosmetic industry, and especially in pharmaceutics [3, 4]. Amidohydrolases may also have useful applications in cancer therapy: anticancer activity has been observed for some of these enzymes [5].

Gene directed enzyme-prodrug therapy (GDEPT) is an auspicious area for amidohydrolase application. GDEPT utilizes a transgene that encodes an enzyme capable of converting a non-toxic prodrug into an active therapeutic metabolite which affects the viability of cancer cells [6]. In this two-step strategy, the gene encoding the non-endogenous enzyme is initially directed towards the tumor tissue. Within the tumor, it undergoes transcription to form mRNA, from which subsequently the therapeutic enzyme is translated inside the cancer cells. This process is then followed by the systemic delivery of the prodrug, which undergoes selective activation into its active drug form only through the action of the foreign enzyme expressed within the cancer cells [7]. This approach relies on precise targeting of cancer cells, while sparing healthy tissue. The therapeutic enzyme’s gene is specifically aimed at cancer cells using various viral vectors or alternative delivery methods [8, 9]. Furthermore, gene expression can be regulated by tumor-specific promoters, enabling the enzyme and its associated enzymatic reaction to be exclusively targeted at tumor cells. This ensures that other cells, even if they uptake the gene and prodrug, remain unaffected [10]. The preferential prodrug conversion can potentially lead to a significantly higher therapeutic index in comparison to conventional cancer chemotherapy drugs.

GDEPT has several clear advantages over traditional cancer therapies: the active cytotoxic drug is produced only in the tumor tissue, which not only increases the success of the therapy but also induces fewer side-effects; also, the prodrug can be administered many times with the same effect while the prodrug activating enzyme is still localized in the tumor, thus achieving complete death of the cancer cells [11]. Furthermore, the utilization of an exogenous enzyme offers the significant advantage of using a prodrug unrecognizable to human enzymes, thereby reducing off-target activity while maximizing toxicity in the tumor environment [7]. To date, several enzyme-prodrug combinations have been developed and shown to have great potential in both pre-clinical studies and early clinical trials, but none of them has reached the clinic due to several drawbacks [12]. Many strategies are hampered by the low specificity of the enzymes for the target prodrugs, high affinity for other natural substrates, limited prodrug uptake, poor pharmacokinetics, and activation of prodrugs by non-target enzymes [13, 14]. The development of new enzyme-prodrug combinations with specific and precise effects is important and necessary to bring GDEPT to the clinic.

Previously, the YqfB [15] and D8_RL [16] proteins were described as small amidohydrolases (103 aa and 128 aa respectively) active towards *N*^4^-acylated cytidines, e.g., *N*^4^-acetylcytidine [15–17]. YqfB is the product of the *yqfB* gene in *Escherichia coli*, while D8_RL was isolated from metagenomic libraries. Both of these enzymes are unique monomeric amidohydrolases belonging to human activating signal cointegrator homology (ASCH) domain-containing proteins, which are widespread and diverse, but so far most of them do not have a confirmed function [18]. Although both YqfB and D8_RL can catalyze the conversion of *N*^4^-acylated cytidines, their structural and catalytic properties are different [16], which makes both enzymes equally interesting objects for this study.

The primary substrate of YqfB, conversion of which is also catalyzed by D8_RL, is *N*^4^-acetylcytidine. This compound is abundant in both prokaryotic and eukaryotic RNA molecules and is essential for efficient protein synthesis as well as for the biogenesis of eukaryotic ribosomes [19, 20]. There are substantial data indicating that various tRNA- and rRNA-specific acetyltransferases are responsible for the formation of *N*^4^-acetylcytidine [21, 22], but its degradation and/or recycling is still not fully understood. However, most of the other known substrates of YqfB and D8_RL, including the ones used in this study, are synthetic compounds. This offers a major advantage for the development of enzyme-prodrug systems: the likelihood that native human enzymes will be able to recognize and activate prodrugs that were developed based on artificial substrates is greatly reduced.

**Fig 1:**
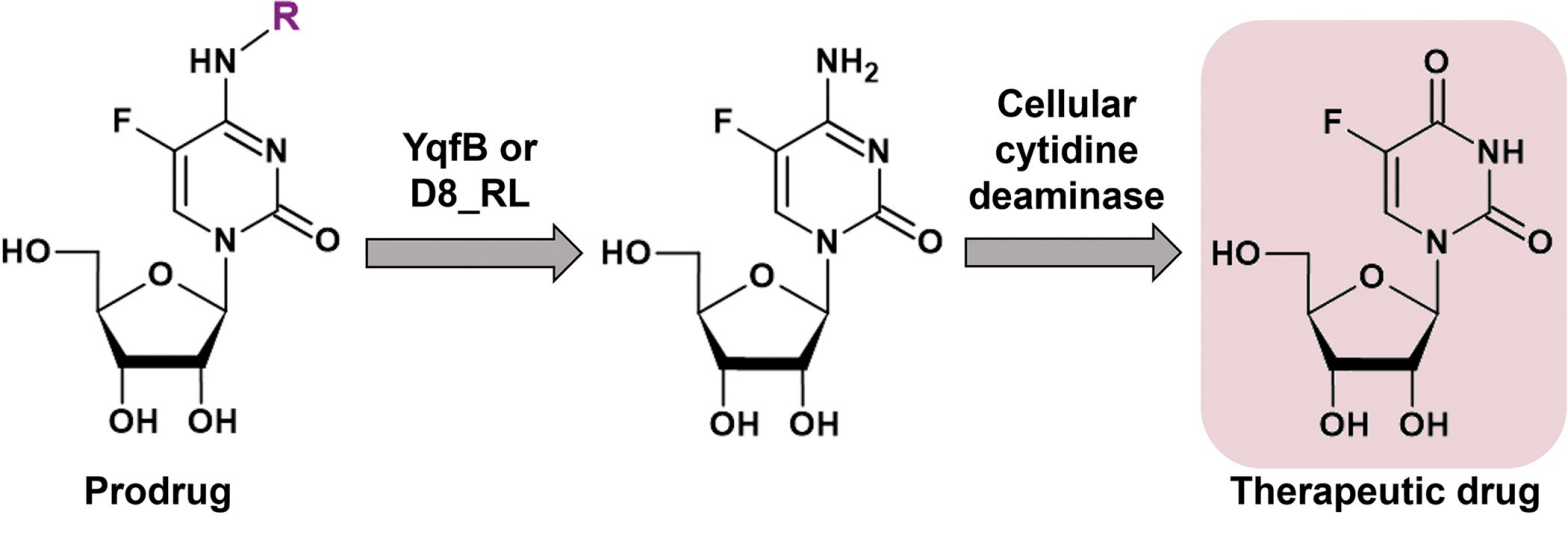
Graphical representation of YqfB/D8_RL enzyme-mediated activation of an anticancer prodrug.

YqfB and D8_RL enzymes have also been observed to hydrolyze capecitabine, a non-toxic form of a well-known chemotherapeutic drug 5-fluorouracil [23]. Once inside the cell, 5-fluorouracil is converted to 5-fluorouridine and 5-fluorodeoxyuridine, which are then processed further by cellular enzymes and become active metabolites that ultimately cause cell death [24]. It is also well known that eukaryotic cells possess cytidine deaminases that can produce 5-fluorouridine and 5-fluorodeoxyuridine from 5-fluorocytidine and 5-fluorodeoxycytidine [25, 26]. Thus, the combination of YqfB or D8_RL amidohydrolase with an intracellular cytidine deaminase could be a promising strategy for the activation of anticancer prodrugs (Fig 1).

Here we describe the potential of YqfB and D8_RL amidohydrolases to be used as prodrug activating enzymes. Several modified 5-fluorocytidine derivatives have been selected for this purpose, thus suggesting a number of enzyme-prodrug combinations potentially suitable for therapeutic applications. We also present data showing that bacterial amidohydrolases YqfB and D8_RL can be active in eukaryotic cell models and that the toxic products generated from the tested prodrugs can affect the viability of these cells.

## Materials and methods

### Chemicals

5-fluorocytidine was purchased from Biosynth Carbosynth (Berkshire, UK). The synthesis of 5-fluoro-*N*^4^-benzoylcytidine and 5-fluoro-*N*^4^-pivaloylcytidine was described previously [17]. The synthesis of 5-fluoro-*N*^4^-acetylcytidine and 5-fluoro-*N*^4^-[3-indolepropionyl]cytidine (Fig A in S1 File) is described in the Supporting information.

### Cell culturing

All procedures were carried out under aseptic conditions meeting biological safety requirements. The human colon cancer cell line HCT116 and the human breast cancer cell line MCF7 were a gift from Dr. Daiva Baltriukienė (Life Sciences Center, Vilnius University, Vilnius, Lithuania). The human embryonal kidney-derived cell line 293FT was obtained from Invitrogen, USA (catalog No. R70007). 293FT cells were maintained in DMEM (Gibco, USA) containing 10% fetal bovine serum (FBS, Gibco, USA), 0.1 mM MEM Non-Essential Amino Acids (NEAA, Gibco, USA), 6 mM L-glutamine (Gibco, USA), 1 mM MEM sodium pyruvate (Gibco, USA), 100 U/ml penicillin and 0.1 mg/ml streptomycin (Gibco, USA), 0.5 mg/ml geneticin (Gibco, USA). HCT116 and MCF7 cell lines were maintained in DMEM containing 10% FBS, 100 U/ml penicillin and 0.1 mg/ml streptomycin. Cells were grown at 37°C with 5% CO_2_ in a water-saturated incubator. For passaging, cells were incubated with trypsin/EDTA (Gibco, USA) at 37°C to detach cells.

### Enzymatic activity measurements and analysis of reaction mixtures

Cloning, expression, and purification of recombinant YqfB and D8_RL enzymes was described previously [15, 16]. The enzymatic reaction mixtures contained a final 5 μM concentration of substrate (5-fluoro-*N*^4^-acetylcytidine, 5-fluoro-*N*^4^-benzoylcytidine, 5-fluoro-*N*^4^-pivaloylcytidine or 5-fluoro-*N*^4^-[3-indolepropionyl]cytidine) and a final 1 μM concentration of the recombinant protein (YqfB or D8_RL) in 25 mM Tris-HCl pH 8 buffer. Reactions were incubated at 37°C for 12 hours. The reaction mixtures were then mixed with an equal part of acetonitrile, centrifuged at 30,000 × *g* for 10 min and 3 μl were analyzed using liquid chromatography-tandem mass spectrometry with a Nexera X2 UHPLC system coupled with LCMS-8050 mass spectrometer (Shimadzu, Japan) equipped with an ESI source used in the negative ionization mode. The chromatographic separation was carried out using a 3 × 150 mm YMC-Triart C18 (particle size 3 μm) column (YMC, Japan) at 40°C and a mobile phase that consisted of 0.1% formic acid (solvent A) and acetonitrile (solvent B) delivered in gradient elution mode at a flow rate of 0.45 ml/min. The following elution program was used: 0 to 1 min, 5% solvent B; 1 to 5 min, 95% solvent B; 5 to 7 min, 95% solvent B; 7 to 8 min, 5% solvent B; 8 to 12 min, 5% solvent B. Mass scans were measured from *m/z* 50 to *m/z* 750 at interface temperature of 300°C and desolvation line temperature of 250°C. N_2_ was used as nebulizing and drying gas, dry air was used as heating gas. LabSolutions LCMS software was used to analyze the occurrence of the hydrolysis product 5-fluorocytidine in the reaction mixtures.

### Preparation of solutions of prodrugs

Solutions of all tested compounds were prepared fresh for each experiment in DMSO diluted in the complete culture medium and added to HCT116 and MCF7 cells to a final concentration of 1–100 μM. The concentration of DMSO in the assay never exceeded 0.02% and had no influence on cell growth.

### MTT assay

HCT116 and MCF7 cells were cultured on 96-well plates at a density of 4000 cells/well. The cells were incubated for 24 hours to allow cells to attach to the culture vessel before they were exposed to several concentrations of prodrugs for 24 hours. After that, culture medium was carefully removed and 100 μl of the MTT solution (0.5 mg/ml MTT reagent (Merck, Germany) in PBS) was added to each well. The metabolically active cells reduced MTT to blue formazan crystals. After 1 hour of incubation at 37°C, MTT-formazan crystals were dissolved in 120 μl DMSO, 100 μl of which was transferred to new 96-well plate suitable for optical measurements. The absorbance of the colored formazan product was measured at 540 and 650 nm by Varioskan Flash Spectrophotometer (Thermo Fisher Scientific, Lithuania). The untreated cells served as negative control group. Prior to performing statistical analysis, the obtained data was processed in three steps: first, the 650 nm measurements (background) were subtracted from each individual 540 nm measurement; the mean measurement value obtained from the samples treated with DMSO was subtracted from control group and each test cell sample group treated with different compounds; viability of the cells was evaluated by comparison of test samples against the negative control and expressed as percentage considering that viability of untreated cells was 100%. Each test group was tested in eight replicates.

### Statistical analysis

All grouped MTT analysis data was presented as mean with 95% confidence interval. Comparisons between groups were made by the one-tailed Welch’s t test for independent samples or one-way ANOVA followed by Tukey’s multiple comparison test. The Shapiro-Wilk test was performed to evaluate deviation from normality. The significance level was chosen at α = 0.05 for all criteria used. Data were plotted and statistical analysis was performed using RStudio version 1.3.1073.

### Plasmid construction

The DNA fragment, encoding His-tagged amidohydrolase YqfB or D8_RL flanked by the unique restriction sites *Bam*HI and *Eco*105I, was cloned into vector pBABE-Puro (Cell Biolabs, Inc., USA) using *Bam*HI and *Eco*105I restriction sites. Genes encoding YqfB and D8_RL (the sequences of *yqfB* and *D8_RL* genes were described in previous publications [11, 12]) were PCR-amplified using 2× Phusion Polymerase Master Mix (Thermo Fisher Scientific, Lithuania) and the following pairs of primers: YqfB_*Bam*HI_FW (5’-TGACGGATCCATGCAGCCAAACGA-3’), YqfB_*Eco*105I_RV (5’-TGACTACGTATCAGTGGTGGTGGTGGTG-3’) for *yqfB* and D8_*Bam*HI_FW (5’-TGACCGGATCCATGGAAC-AATTAAAATTTC-3’), D8_*Eco*105I_RV (5’-TGACTACGTATTAGCCGTGGTGGTGATG-3’) for *D8_RL*. The PCR products were digested with *Bam*HI and *Eco*105I (Thermo Fisher Scientific, Lithuania) and ligated to pBABE-Puro using T4 DNA Ligase (Thermo Fisher Scientific, Lithuania) according to instructions of the manufacturer. Constructs were sequence-verified by Macrogen Europe (Amsterdam, Netherlands).

### Retroviral generation of stable cell lines

Retroviral pBABE-Puro vector encoding the desired construct (1066.6 ng) were co-transfected with pCMV-gag-pol (355.6 ng) and pCMV-VSV-G (177.8 ng) (Cell Biolabs, Inc., USA) into 4 cm^2^ size dish of ~70% confluent 293FT cells. 100 μl Opti-MEM medium (Gibco, USA) was mixed with 4 μl Lipofectamine 2000 reagent (Invitrogen, USA) and incubated for 5 minutes at room temperature. Then, plasmids diluted in 100 μl Opti-MEM medium were added to the diluted Lipofectamine 2000. Following a gentle mix and incubation at room temperature for 20 minutes, the transfection mix was added dropwise to 293FT cells. 16 hours post-transfection, fresh medium was added to the cells. 24 hours later, the retroviral medium was collected and passed through 0.45 μm sterile syringe filters. Target HCT116 and MCF7 cells (∼60% confluent) were transduced with the optimized titre of the retroviral medium diluted in fresh medium (typically 1:1–1:10) containing 8 μg/ml polybrene (Sigma-Aldrich, USA) for 24 hours. The retroviral medium was then replaced with fresh medium, and 24 hours later, the medium was again replaced with fresh medium containing 2 μg/ml puromycin (Sigma-Aldrich, USA) for selection of cells which had integrated the construct. A pool of transduced cells was utilized for subsequent experiments following complete death of non-transduced cells placed under selection in parallel.

### Expression analysis of mRNA

Total RNA was extracted from transduced HCT116 and MCF7 cell lines using TRIzol Reagent (Thermo Fisher Scientific, Vilnius, Lithuania) and the cDNA synthesis was performed with Maxima H Minus First Strand cDNA Synthesis Kit with dsDNase (Thermo Fisher Scientific, Vilnius, Lithuania), according to the manufacturer’s instructions. The mRNA expressions of *yqfB* and *D8_RL* were analyzed by Real-Time Quantitative PCR (RT-qPCR) using Luminaris Color HiGreen High ROX kit (Thermo Fisher Scientific, Lithuania). The primers were used as follows: YqfB_qPCR_FW (5’-ACATTCTGGCTGGGCGTAAA-3’), YqfB_qPCR_RV (5’-ATCCAGCGTTACGGTTGAGG-3’) for *yqfB* (149 bp amplicon size) and D8_qPCR_FW (5’-GGTCCGAAGTACACGGTTGG-3’), D8_qPCR_RV (5’-AGGCGA-CTGATCCATTCGTT-3’) for *D8_RL* (137 bp amplicon size). In each set of RT-qPCR analyses, two negative controls were used: nuclease-free water and cells expressing ‘‘empty” control vector pBABE-Puro. Amplification parameters for both the *yqfB* and the *D8_RL* were those offered by the manufacturer.

## Results

### *In vitro* screening of potential prodrugs

**Fig 2:**
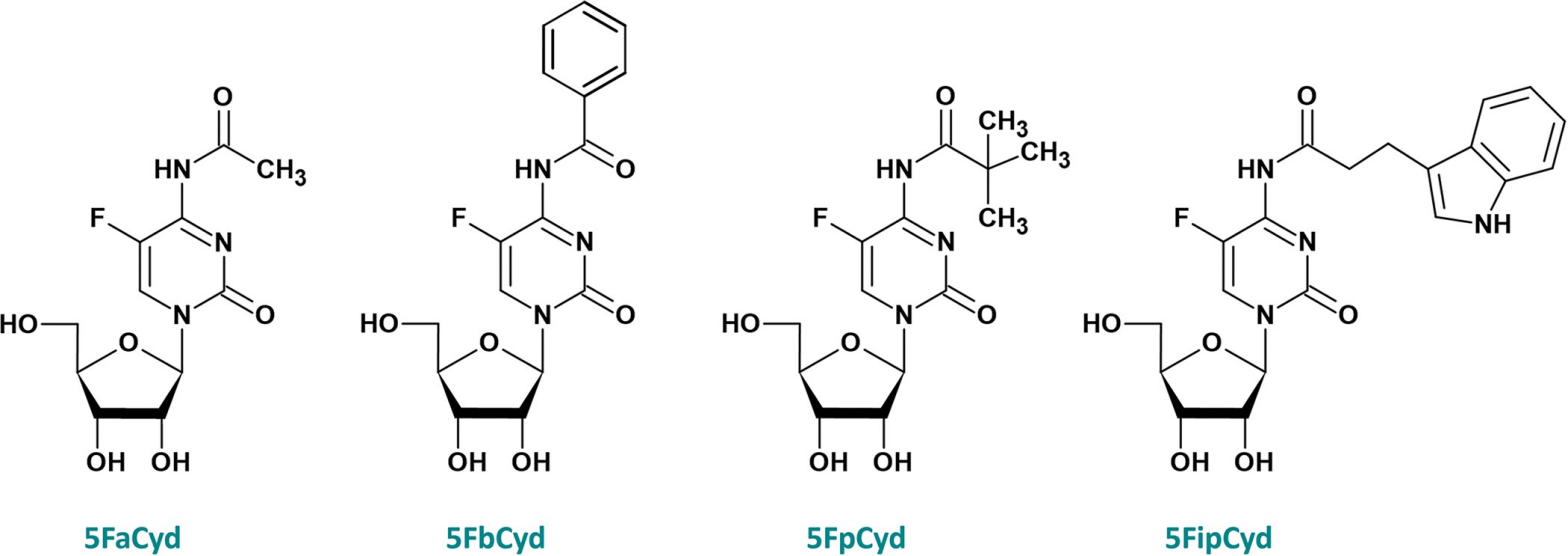
Substrates of YqfB and D8_RL amidohydrolases. 5FaCyd– 5-fluoro-*N*^4^-acetylcytidine, 5FbCyd– 5-fluoro-*N*^4^-benzoylcytidine, 5FpCyd– 5-fluoro-*N*^4^-pivaloylcytidine, 5FipCyd– 5-fluoro-*N*^4^-[3-indolepropionyl]cytidine.

In this study, we aimed to investigate which prodrugs are potential substrates for YqfB and D8_RL amidohydrolases. Several *N*^4^-acylated 5-fluorocytidine derivatives (Fig 2) were incubated with purified enzymes and, after 12 hours of incubation, samples were analyzed by LC-MS.

**Fig 3:**
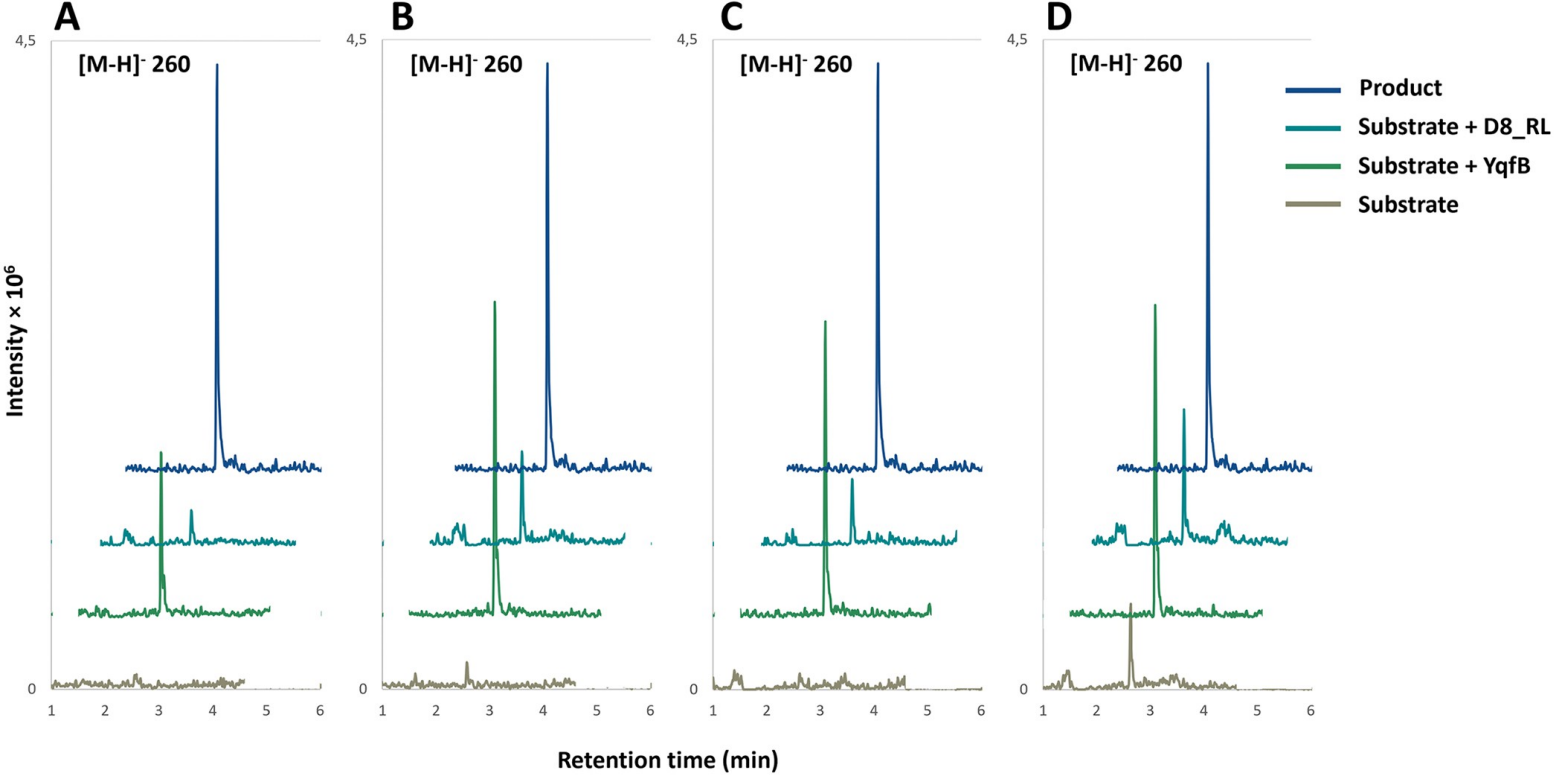
Substrate specificity of YqfB and D8_RL amidohydrolases. The graphs show mass traces of the 5-fluorocytidine (*m/z* [M-H]^-^ 260) in the samples from the enzymatic reactions. YqfB and D8_RL amidohydrolases were incubated with (A) 5-fluoro-*N*^4^-acetylcytidine, (B) 5-fluoro-*N*^4^-benzoylcytidine, (C) 5-fluoro-*N*^4^-pivaloylcytidine, and (D) 5-fluoro-*N*^4^-[3-indolepropionyl]cytidine. The product 5-fluorocytidine was used as a positive control (highlighted in dark blue).

As expected, both YqfB and D8_RL amidohydrolases are able to hydrolyze 5-fluoro-*N*^4^-acetylcytidine to 5-fluorocytidine (Fig 3), since *N*^4^-acetylcytidine is their primary substrate. In addition, the enzymes were active towards all other substrates tested: 5-fluoro-*N*^4^-benzoylcytidine, 5-fluoro-*N*^4^-pivaloylcytidine and 5-fluoro-*N*^4^-[3-indolepropionyl]cytidine.

The recombinant D8_RL enzyme displayed a lower *in vitro* efficiency compared to the recombinant YqfB enzyme, and this should be taken into consideration in further studies regarding the design of experimental conditions. However, both enzymes are suitable for further analysis as even lower amounts of the product generated can lead to biologically significant changes in cell viability.

### Effect of selected prodrugs on the viability of cancer cell lines

**Fig 4:**
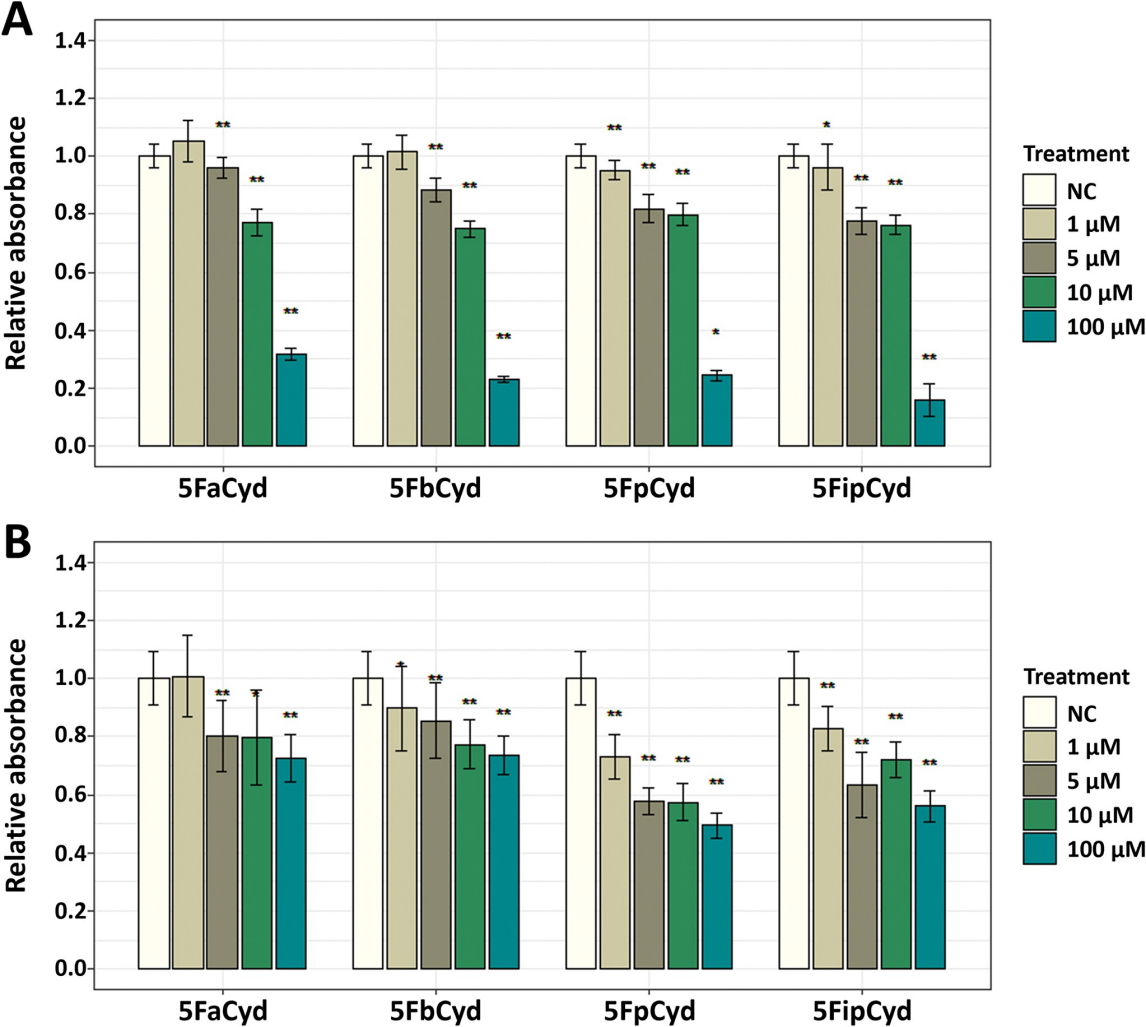
MTT assay of HCT116 (A) and MCF7 (B) cell lines following treatment of prodrugs. Both cell lines were exposed to concentrations of 1–100 μM of the target prodrugs for 24 hours. 5FaCyd– 5-fluoro-*N*^4^-acetylcytidine, 5FbCyd– 5-fluoro-*N*^4^-benzoylcytidine, 5FpCyd– 5-fluoro-*N*^4^-pivaloylcytidine, 5FipCyd– 5-fluoro-*N*^4^-[3-indolepropionyl]cytidine. Statistical significance indicated by *p*-values, where the symbol * designates *p* < 0.05, whereas the symbol ** designates *p* < 0.01 with respect to untreated cells (negative control (NC)).

To investigate whether the selected prodrugs do not exert toxic effects on eukaryotic cells prior to their activation, HCT116 and MCF7 cancer cell lines were treated with several concentrations of the target compounds. Cell viability was assessed by MTT assay 24 hours after the exposure (Fig 4).

At lower concentrations of all tested prodrugs (1–10 μM), the viability of HCT116 cells was not biologically significantly different compared to untreated control cells. However, at a concentration of 100 μM, cell viability was significantly reduced, and further studies should avoid concentrations above 10 μM for these cells. MCF7 cells highlighted a slightly different response. Each prodrug individually showed a similar decrease in cell viability across the range of concentrations tested. 5-fluoro-*N*^4^-pivaloylcytidine and 5-fluoro-*N*^4^-[3-indolepropionyl]cytidine showed higher toxicity to MCF7 cells compared to 5-fluoro-*N*^4^-acetylcytidine and 5-fluoro-*N*^4^-benzoylcytidine. However, the different results obtained between the different cell lines suggest that all the selected prodrugs should be studied further.

### Generation of stable cell lines expressing bacterial amidohydrolases

**Fig 5:**
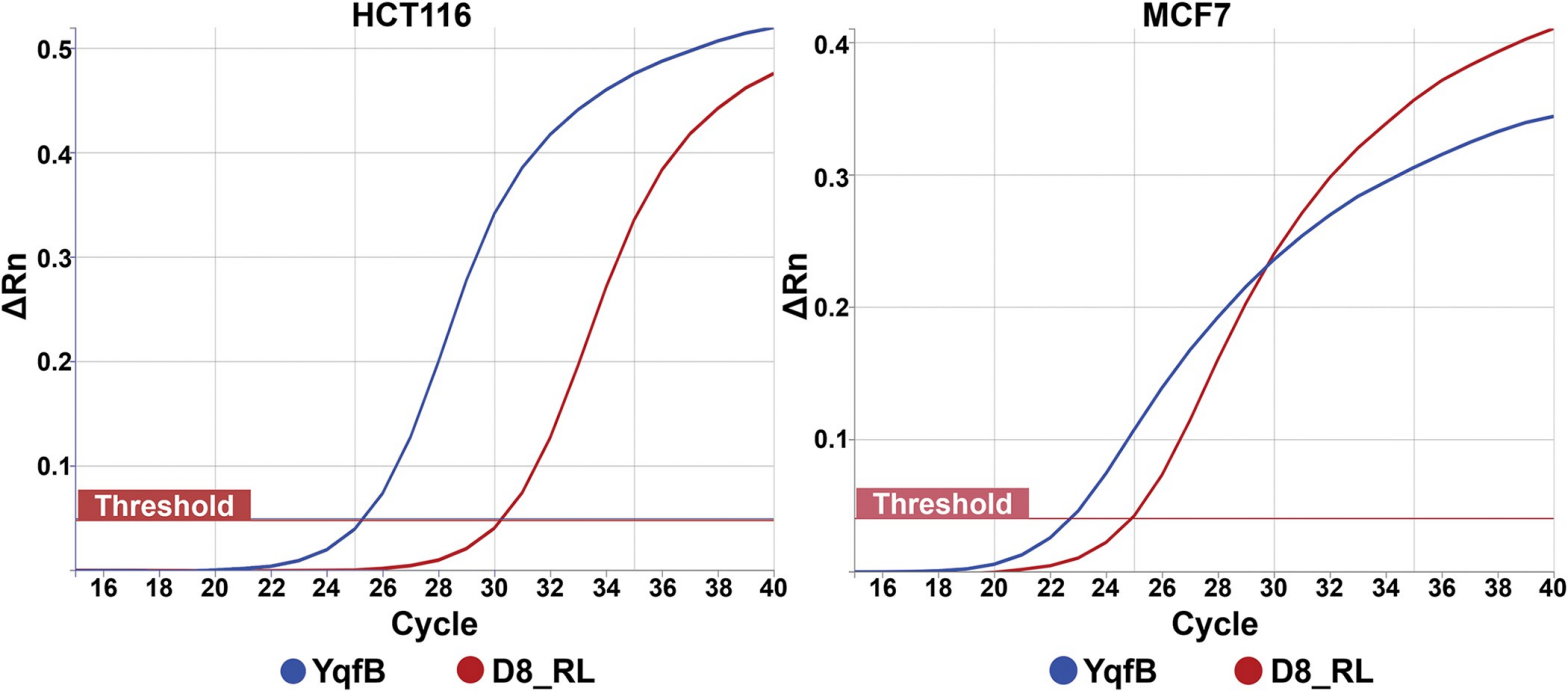
Confirmation of *yqfB* and *D8_RL* expression in HCT116 and MCF7 cells by real time PCR. Amplification plots for different transduced cell lines displayed (y axis (ΔRn)–normalized fluorescence of SYBR Green probe, x axis–PCR cycle number). Exponential increase of fluorescence with values crossing the threshold confirm the presence of *yqfB* and *D8_RL* mRNA in the corresponding transduced cell lines.

Further, we aimed to find out whether bacterial amidohydrolases can activate prodrugs in eukaryotic cells. Cell lines stably expressing YqfB or D8_RL amidohydrolases were generated for this purpose. Genes encoding YqfB and D8_RL enzymes were inserted into the genome of both HCT116 and MCF7 cancer cell lines by retroviral transduction. The expression of the inserted genes in the generated cell lines was confirmed by real time PCR (Fig 5).

To confirm YqfB and D8_RL gene expression at protein level, Western blot analysis was performed on protein extracts prepared from both cell lines expressing YqfB and D8_RL amidohydrolases (S2 File). We hypothesize that the level of these proteins in the cells is too low for detection using this method. The minimum threshold that can be detected via Western blotting with the primary antibodies against 6x-His Tag is 25 ng of protein per sample (Fig A in S2 File). Therefore, we cross-checked the abundance of YqfB and D8_RL proteins in our cell lines using LC-MS/MS proteomic analysis (S3 File). The YqfB protein was detected in the HCT116 cell line when the 6×His-tagged proteins in the extract sample were enriched 8-fold (Fig A in S3 File). The sequence coverage of YqfB was 44–78%. The concentration of this protein in HCT116 cell extract was estimated to be approximately 10.3 ng/ml, which confirms that the quantity of recombinant protein is below the detection limit of Western blot assay. Nevertheless, the proteomic analysis confirmed the presence of YqfB in the HCT116 cell line at protein level. In addition, the catalytic parameters determined for YqfB and D8_RL enzymes [15, 16] demonstrated high enzymatic activities. Therefore, it is to be expected that these enzymes have a biological effect even in very low concentrations, which makes their therapeutic efficacy highly feasible.

### Hydrolysis of prodrugs in eukaryotic cells by YqfB and D8_RL enzymes

**Fig 6:**
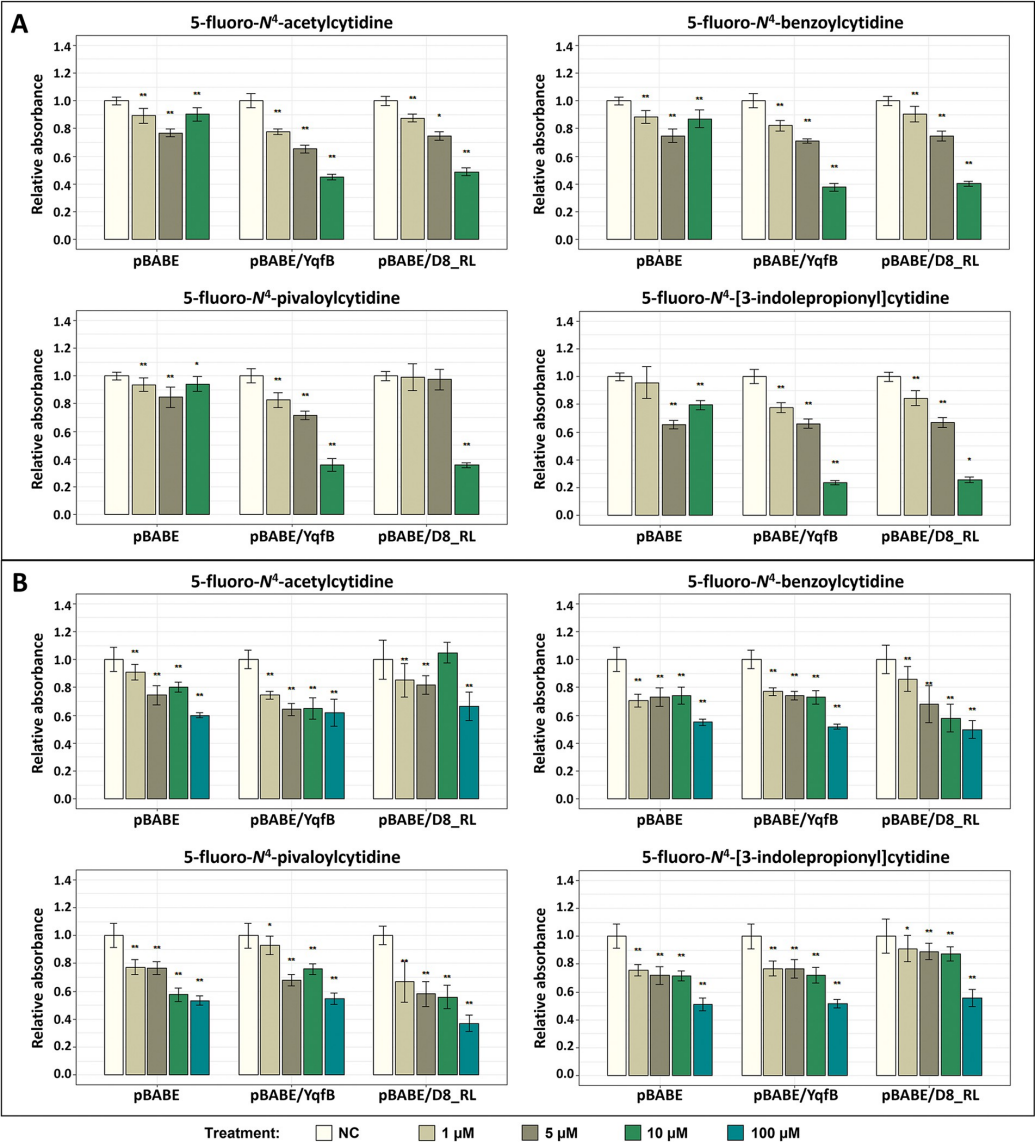
MTT assay of cell lines expressing YqfB and D8_RL following treatment of prodrugs. HCT116 (A) and MCF7 (B) cell lines transduced with YqfB-encoding vector pBABE/YqfB, D8_RL-encoding vector pBABE/D8_RL or control vector pBABE-Puro (designated as pBABE) were exposed to several different concentrations (1–10 μM for HCT116 and 1–100 μM for MCF7) of prodrug 5-fluoro-*N*^4^-acetylcytidine, 5-fluoro-*N*^4^-benzoylcytidine, 5-fluoro-*N*^4^-pivaloylcytidine, and 5-fluoro-*N*^4^-[3-indolepropionyl]cytidine for the 24 hours. Statistical significance indicated by *p*-values, where the symbol * designates *p* < 0.05, whereas the symbol ** designates *p* < 0.01 with respect to untreated cells (negative control (NC)).

We aimed to assess whether the bacterial amidohydrolases YqfB and D8_RL have the potential to be used in enzyme-prodrug therapy. To achieve this, we analyzed whether the target enzymes could activate prodrugs inside the cancer cells leading to a decrease in their viability. The HCT116 and the MCF7 cell lines stably expressing YqfB or D8_RL amidohydrolases were treated with several different concentrations of the selected prodrugs (1–10 μM for HCT116 and 1–100 μM for MCF7). Cell viability was assessed by MTT assay 24 hours after the exposure and compared to control cells transduced with a vector without any gene insert (Fig 6).

The viability of HCT116 cell lines expressing the YqfB or D8_RL amidohydrolase genes was significantly reduced after exposure to all tested prodrugs. Differences in cell viability between amidohydrolase-expressing and control cells were most clearly observed when cells were exposed to 10 μM of the prodrugs. These results indicate that bacterial amidohydrolases convert the non-toxic prodrugs into the therapeutic agent 5-fluorouridine in the HCT116 cell line when acting together with cellular cytidine deaminase. These results are supported by the fact that cell viability is similarly reduced by both 5-fluorocytidine and 5-fluorouridine (S1 Fig). However, the viability of MCF7 cell lines with inserted YqfB or D8_RL genes did not differ from the control cells for all prodrugs tested. This suggests that although YqfB and D8_RL amidohydrolases in combination with modified cytidine-based prodrugs could serve as novel enzyme-prodrug systems, the success of the therapy may depend on the type and/or origin of the cancer cells.

### Effect of hydrolysis products derived from the complex prodrug 5-fluoro-*N*^4^-[3-indolepropionyl]cytidine on the viability of HCT116 cells

**Fig 7:**
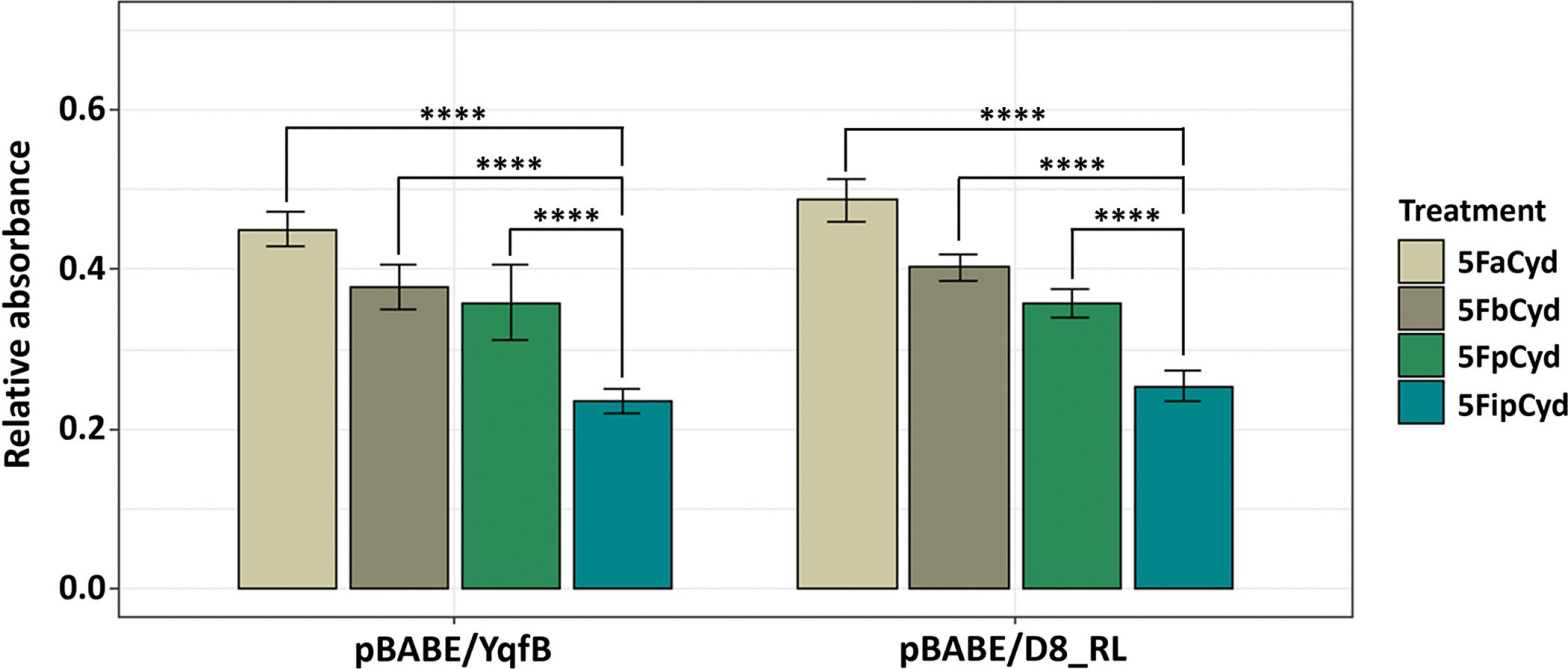
MTT assay of the HCT116 cell lines expressing YqfB or D8_RL after exposure to 10 μM of prodrugs. The HCT116 cell line transduced with a YqfB-encoding vector is designated as pBABE/YqfB and the one transduced with a D8-encoding vector is designated as pBABE/D8_RL. 5FaCyd– 5-fluoro-*N*^4^-acetylcytidine, 5FbCyd– 5-fluoro-*N*^4^-benzoylcytidine, 5FpCyd– 5-fluoro-*N*^4^-pivaloylcytidine, 5FipCyd– 5-fluoro-*N*^4^-[3-indolepropionyl]cytidine. Significant differences in cell viability were obtained between cells treated with different prodrugs when one-way ANOVA test (Tukey’s multiple comparison) was performed (****p < 0.0001).

While analyzing the effects of the activated prodrugs on the viability of the HCT116 cell line we observed differences in toxicity regardless of the fact that the cells were treated with the same concentrations of the tested compounds. Both YqfB and D8_RL amidohydrolase-expressing HCT116 cells showed the most significant decrease in viability after exposure to 5-fluoro-*N*^4^-[3-indolepropionyl]cytidine (Fig 7). This led to the analysis of possible factors contributing to the higher toxicity of this substrate.

**Fig 8:**
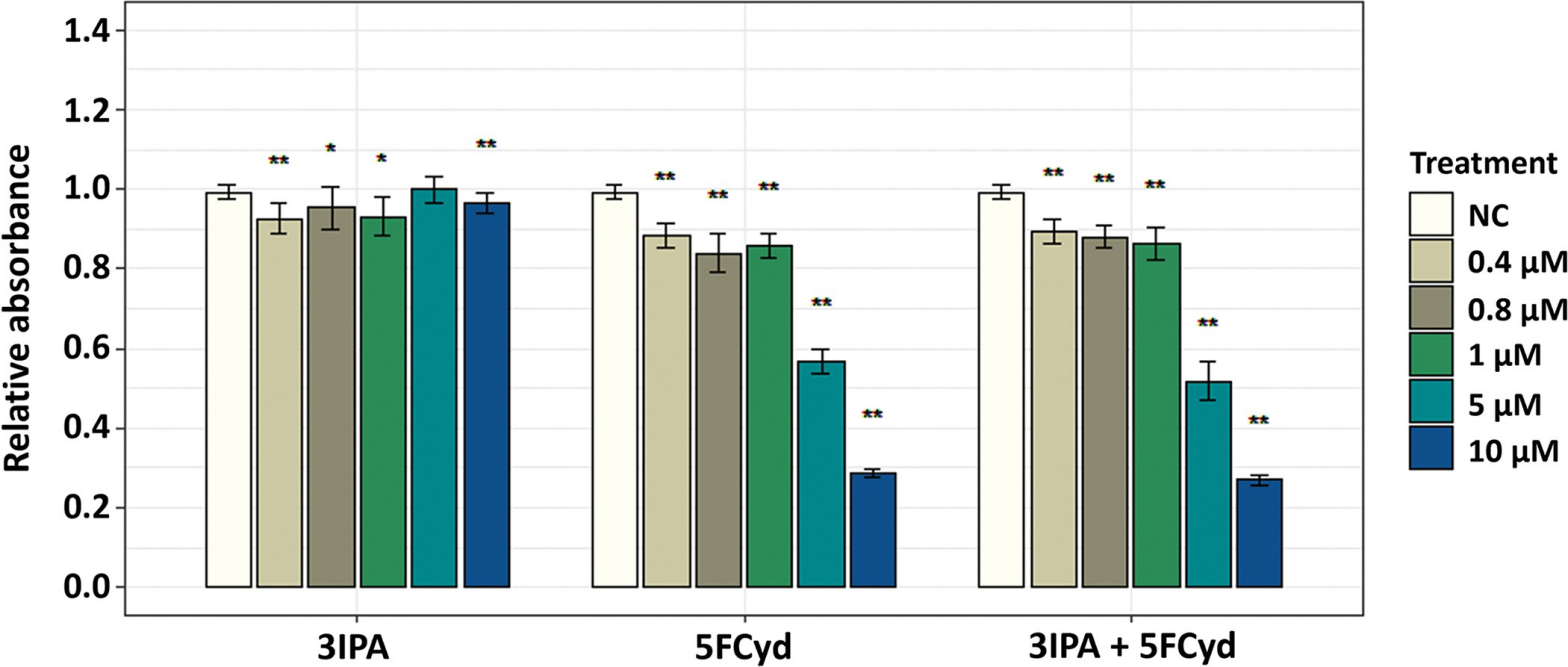
MTT assay of HCT116 cell line following treatment of hydrolysis products derived from the complex prodrug 5-fluoro-*N*^4^-[3-indolepropionyl]cytidine. The HCT116 cell line was exposed to concentrations of 0.4–10 μM of 5-fluorocytidine (5FCyd), 3-indolepropionic acid (3IPA) and a mixture of these two compounds for 24 hours. Statistical significance indicated by *p*-values, where the symbol * designates *p* < 0.05, whereas the symbol ** designates *p* < 0.01 with respect to untreated cells (negative control (NC)).

Hydrolysis of 5-fluoro-*N*^4^-[3-indolepropionyl]cytidine by YqfB or D8_RL enzyme produces not only 5-fluorocytidine but also a second product– 3-indolepropionic acid. We hypothesized that it might have an additional toxic effect on cancer cells. 3-indolepropionic acid was shown to have cytostatic and antineoplastic properties in breast cancer [27], hence we decided to test whether a similar effect could be observed in our target cells. HCT116 cells were treated with different concentrations of 3-indolepropionic acid, 5-fluorocytidine and a mixture of these compounds and cell viability was assessed by MTT after 24 hours (Fig 8).

The results showed that a combination of 5-fluorocytidine and 3-indolepropionic acid had nearly the same toxic effect on HCT116 cells as 5-fluorocytidine alone. No biologically significant effect of 3-indolepropionic acid on cell viability was observed. Although the literature suggests that 3-indolepropionic acid has anticancer properties, we did not obtain a similar result. The origin of the cells used in our study differs from the previously described breast cancer cells and this may have contributed to the fact that we did not observe the same result. 5-fluoro-*N*^4^-[3-indolepropionyl]cytidine may have higher toxicity due to any number of factors that would be worth investigating. Testing the effects of this prodrug on multiple cancer cell lines could be a starting point for further research. Another important consideration is that the hydrolysis of this substrate produces two products in equal proportions, and further studies should therefore consider which concentrations of both products are appropriate to achieve the maximum toxic effect on cancer cell lines.

## Discussion

It is known from previous studies that various amidohydrolases due to their physicochemical properties are attractive objects in many fields and have potential applications in food and pharmaceutical industries as well as in biotechnology [28–30]. Here, we have demonstrated that amidohydrolases have the potential to be applied in GDEPT and used for therapeutic purposes. We also suggest four novel prodrug variants suitable for hydrolysis by the YqfB and D8_RL enzymes, which indicates that these amidohydrolases could be used with an even wider range of potential prodrugs. This allows for the design of several enzyme-prodrug combinations, which increases the chances that at least one of the systems will be suitable for future use in the clinic. While analyzing the toxicity of our prodrugs towards various cancer cells, we found that some of these compounds were slightly toxic to MCF7 breast cancer cell line. Advantageously, the broad range of amidohydrolase substrates will allow to search for alternative options and reduce the likelihood of toxicity of the prodrugs prior to their activation. In addition, the YqfB and D8_RL enzymes are relatively small (12 kDa and 15 kDa respectively), which may be particularly useful for clinical applications, especially considering the transfer of DNA fragments. To the best of our knowledge, some of the most efficient gene transfer systems are still virus-based and one of the main drawbacks of this strategy is the limited packaging capacity [31]. Nevertheless, this should not affect the transfer of genes encoding YqfB and/or D8_RL (312 bp and 387 bp respectively). Moreover, the halotolerance of these enzymes opens up the possibility of delivering them into the tumor microenvironment as a protein that is likely to remain stable and active even in biological fluids such as blood. In a recent study, analogues of YqfB amidohydrolase found in other microorganisms showed better catalytic efficiency compared to YqfB for the primary substrate *N*^4^-acetylcytidine [17]. This suggests that more studies may lead to even better amidohydrolase enzyme-prodrug combinations to achieve the highest therapeutic efficacy.

In the present work, HCT116 and MCF7 cancer cell lines stably expressing YqfB and D8_RL amidohydrolases were generated. Previous studies have used genes encoding human-optimized versions of bacterial enzymes [32], but we have shown here that expression of non-optimized genes of the bacterial amidohydrolases occurs in eukaryotic cells. In HCT116 cells expressing both YqfB and D8_RL, we observed a statistically significant decrease in cell viability when exposed to selected prodrugs, indicating that these cells synthesize active amidohydrolases capable of converting the target compounds to 5-fluorocytidine, which is subsequently converted by a cellular cytidine deaminase into its toxic form. This demonstrates that YqfB and D8_RL amidohydrolases, in combination with *N*^4^-acylated 5-fluorocytidine derivatives, could be applied as novel enzyme-prodrug systems in GDEPT. To further confirm this, future studies should assess the success of the suggested enzyme-prodrug combinations using more complex systems, e.g., organotypic cultures.

We also report data showing that the viability of amidohydrolase-encoding MCF7 cells after exposure to all the tested prodrugs did not differ from that of control cells. There may be two reasons for this: either the proteins synthesized in the cells are inactive, or the prodrugs tested are excessively toxic to the MCF7 cell line and we were no longer able to observe a difference between the control and the amidohydrolase-encoding cells. There is also the possibility that the selected enzyme-prodrug pairs may not be suitable for breast cancer therapy, as there have already been studies showing that different characteristics of cancer cell lines lead to differences in sensitivity to certain therapeutic agents [33]. These results highlight the need for further studies using other cancer cell lines to analyze the success of amidohydrolase-based enzyme-prodrug therapy under different conditions. This would also provide a better understanding of whether the system we propose is universal, or if it could only be applied to the treatment of a specific cancer type.

Ultimately, the enzyme-prodrug system we present has the advantage of versatile prodrug design, which could allow the production of two separate toxic compounds right after hydrolysis of one substrate. This feature, with the appropriate combination of compounds, would allow to reduce the concentration of the prodrug administered into the body while achieving the same or even greater toxic effect, thus increasing the therapeutic applicability of the system. There are several examples in the literature where a single prodrug can be used to produce two toxic compounds that act synergistically or even have different biological effects [34, 35]. One of the four prodrugs we have investigated, 5-fluoro-*N*^4^-[3-indolepropionyl]cytidine, also has this characteristic: both products obtained after hydrolysis– 5-fluorocytidine and 3-indolepropionic acid–are potential anticancer agents [27]. We aimed to show that their cumulative toxicity on cancer cells is greater than that of each of them individually, however, the results obtained using the HCT116 cells showed that 3-indolepropionic acid does not affect the viability of this cell line and the cumulative effect of the compounds is approximately the same as that of 5-fluorocytidine alone. Nevertheless, the possibility of masking the effect of the two compounds in a single prodrug in our proposed system remains and may be one of the most desirable features for future studies using different cancer cell lines.

## Supporting information

S1 File General procedure for the synthesis of *N*^4^-acylated nucleosides. (DOCX)

S2 File Western blot analysis of YqfB and D8_RL. (DOCX)

S3 File Proteomic analysis of YqfB and D8_RL. (DOCX)

S1 Fig MTT assay of MCF7 and HCT116 cell lines following treatment of control compounds. Both cell lines were exposed to concentrations of 1–100 μM of the target compounds for 24 hours. 5FCyd– 5-fluorocytidine, 5FUR– 5-fluorouridine. Statistical significance indicated by p-values, where the symbol * designates p < 0.05, whereas the symbol ** designates p < 0.01 with respect to untreated cells (negative control (NC)).
(TIF)
