# Supplementary material for: Bacterial amidohydrolases and modified 5-fluorocytidine compounds: Novel enzyme-prodrug pairs
Source: PLoS One. 2023 Nov 30;18(11):e0294696. doi: 10.1371/journal.pone.0294696 (PMC10688628; doi:10.1371/journal.pone.0294696)
Supplement: S1 File — (DOCX) [file pone.0294696.s001.docx]

**Supporting information**

**General procedure for the synthesis of N^4^-acylated nucleosides (3a, 3b)**

Appropriate carboxylic acid (**1a**, **1b**) (5.75 mmol) was dissolved in 40 mL of ethyl acetate, *N*-hydroxysuccinimide (NHS, 727 mg, 6.325 mmol) and *N*,*N*′-dicyclohexylcarbodiimide (DCC, 1.305 g, 6.325 mmol) were added, and the mixture was stirred at room temperature for 24 h to produce activated carboxylic acid (**2a**, **2b**). The formed precipitate was removed by filtering, and the solvent was evaporated under reduced pressure. The activated acid was dissolved in 10 mL of dimethylformamide (DMF), 5-fluorocytidine (1 g, 3.83 mmol) was added, and the reaction mixture was stirred at room temperature for 5–7 days. The reaction was monitored by thin-layer chromatography (TLC, chloroform/methanol, 5:1). DMF was removed from the mixture using a rotary evaporator. The product (**3a**, **3b**) was purified by column chromatography (silica gel, chloroform/methanol, 100:0 to 80:20). The final nucleosides were obtained in 5–28% yields. The structures were confirmed by HPLC-MS and NMR analyses.

**
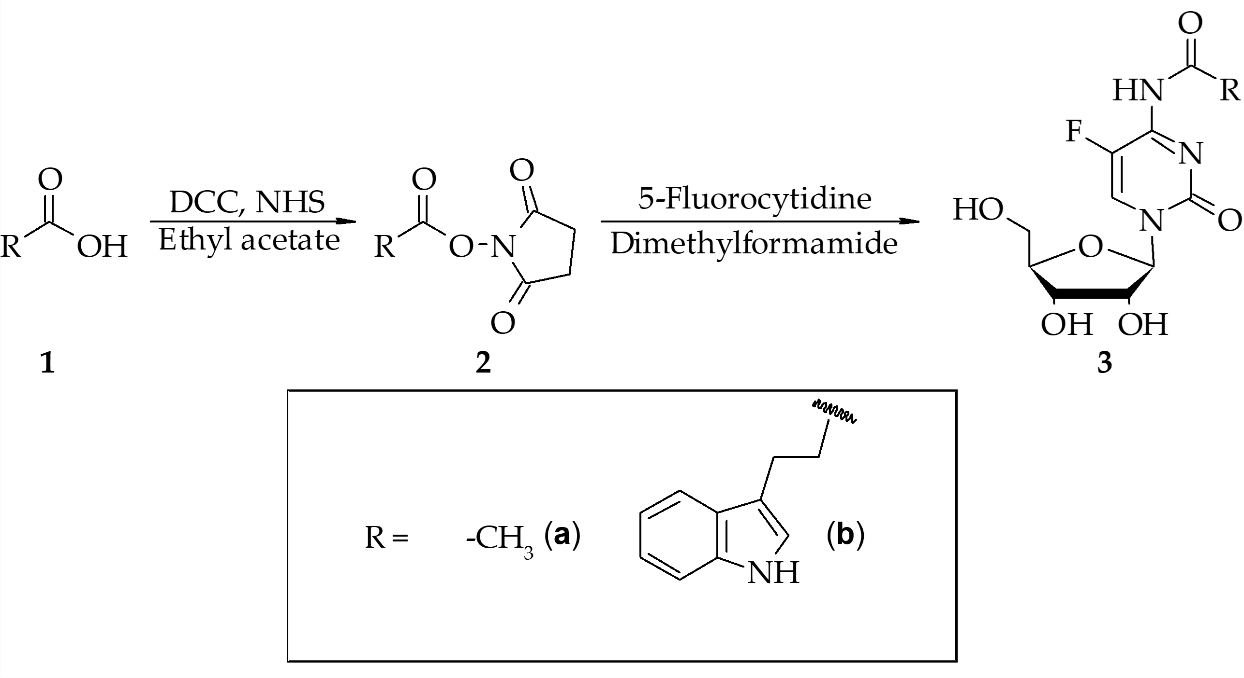
Fig A.** **Synthesis of *N*^4^-acylated nucleosides.**

***N*-(1-((2*R*,3*R*,4*S*,5*R*)-3,4-Dihydroxy-5-(hydroxymethyl)tetrahydrofuran-2-yl)-5-fluoro-2-oxo-1,2-dihydropyrimidin-4-yl)acetamide (3a)**

Yield 320 mg (28%), yellow solid. MS (ESI^+^): m/z 303.75 [M+H]^+^, 301.90 [M-H]^–^. UV λ_max_ 244; 310 nm.

^1^H NMR (DMSO-d_6_, 400 MHz): δ = 2.08 (s, 3H, CH_3_), 3.59 (s, 1H, CH), 3.64 (s, 1H, CH), 3.96–4.07 (m, 1H, CH), 4.18 – 4.30 (m, 2H, CH_2_), 5.01–5.16 (m, 1H, CH), 5.68–5.74 (m, 1H, OH), 5.80 (dd, *J* = 1.9, 5.7 Hz, 1H, OH), 5.93 (dd, *J* = 1.8, 4.8 Hz, 1H, OH), 8.14 (d, *J* = 7.1 Hz, 1H, CH), 9.41 (s, 1H, NH).

^13^C NMR (DMSO-d_6_, 101 MHz): δ = 21.22, 61.01, 72.72, 82.53, 89.17, 125.71, 135.52, 154.16, 157.83, 170.25, 173.35.

***N*-(1-((2*R*,3*R*,4*S*,5*R*)-3,4-Dihydroxy-5-(hydroxymethyl)tetrahydrofuran-2-yl)-5-fluoro-2-oxo-1,2-dihydropyrimidin-4-yl)-3-(1*H*-indol-3-yl)propanamide (3b)**

Yield 90 mg (5%), brown residue. MS (ESI^+^): m/z 432.80 [M+H]^+^, 431.05 [M-H]^–^. UV λ_max_ 247; 291 nm.

^1^H NMR (DMSO-d_6_, 400 MHz): δ = 2.86 – 2.95 (m, 2H, CH_2_), 2.99 (dd, *J* = 8.2, 5.8 Hz, 2H, CH_2_), 3.56 – 3.67 (m, 1H, CH), 3.73 – 3.87 (m, 1H, CH), 3.87 – 3.94 (m, 1H, CH), 3.99 (s, 2H, CH_2_), 4.11 (d, *J* = 5.3 Hz, 1H, CH), 4.90 – 5.15 (m, 1H, OH), 5.36 (d, *J* = 4.9 Hz, 1H, OH), 5.70 (d, *J* = 1.8 Hz, 1H, OH), 6.98 (ddd, *J* = 7.9, 6.9, 1.1 Hz, 1H, CH), 7.07 (ddd, *J* = 8.2, 7.0, 1.2 Hz, 1H, CH), 7.13 (d, *J* = 2.3 Hz, 1H, CH), 7.33 (d, *J* = 8.1 Hz, 1H, CH), 7.57 (d, *J* = 7.8 Hz, 1H, CH), 8.70 (d, *J* = 6.8 Hz, 1H, CH), 10.64 (s, 1H, NH), 10.76 – 10.83 (m, 1H, NH).

^13^C NMR (DMSO-d_6_, 101 MHz): δ = 20.60, 25.69, 49.07, 59.85, 68.67, 69.45, 74.99, 84.53, 89.73, 90.79, 111.79, 113.77, 118.66, 118.83, 121.39, 121.83, 127.43, 136.69, 170.72, 173.24.
