# Supplementary material for: Bacterial amidohydrolases and modified 5-fluorocytidine compounds: Novel enzyme-prodrug pairs
Source: PLoS One. 2023 Nov 30;18(11):e0294696. doi: 10.1371/journal.pone.0294696 (PMC10688628; doi:10.1371/journal.pone.0294696)
Supplement: S2 File — (DOCX) [file pone.0294696.s002.docx]

**Supporting information**

**Western blot analysis of YqfB and D8_RL**

**Method A: Whole-cell extract preparation and Western blot analysis**

Whole-cell extracts of HCT116 and MCF7 cell lines expressing YqfB or D8_RL amidohydrolases were prepared by resuspending the cell pellet in RIPA lysis buffer (50 mM Tris-HCl; 1.5 M NaCl, pH 7.6; 1% Triton X-100; 5 mM sodium dodecyl sulphate; 25 mM sodium deoxycholate; 1% phenylmethylsulfonyl fluoride, 0.2% aprotinin, 0.2% sodium orthovanadate). The extracts were cleared by centrifugation for 15 min at 12000× g at 4 °C. Subsequently, total extract proteins were fractionated by 14% SDS-PAGE and transferred onto the nitrocellulose membranes. After the blocking for 1 hour in 2% bovine serum albumin dissolved in Tris-buffered saline (TBS), the immobilized proteins were incubated overnight at 4 °C with the primary mouse monoclonal antibody against 6x-His Tag (Thermo Fisher Scientific, Lithuania; catalog No. MA1-21315, dilution 1:1000) in blocking solution. After extensive washing in TBS-T buffer (TBS supplemented with 0.05% Tween-20), membranes were incubated with the horseradish peroxidase- (HRP-) conjugated anti-mouse secondary antibody (Carl Roth, Germany; catalog No. 4759, dilution 1:10000) for 1 hour at 22 °C. Immunocomplexes were visualized using an enhanced chemiluminescence substrate (Pierce ECL Western Blotting Substrate, Thermo Fisher Scientific, Lithuania) and documented by Uvitec Alliance imaging system (Uvitec Cambridge, United Kingdom).

**
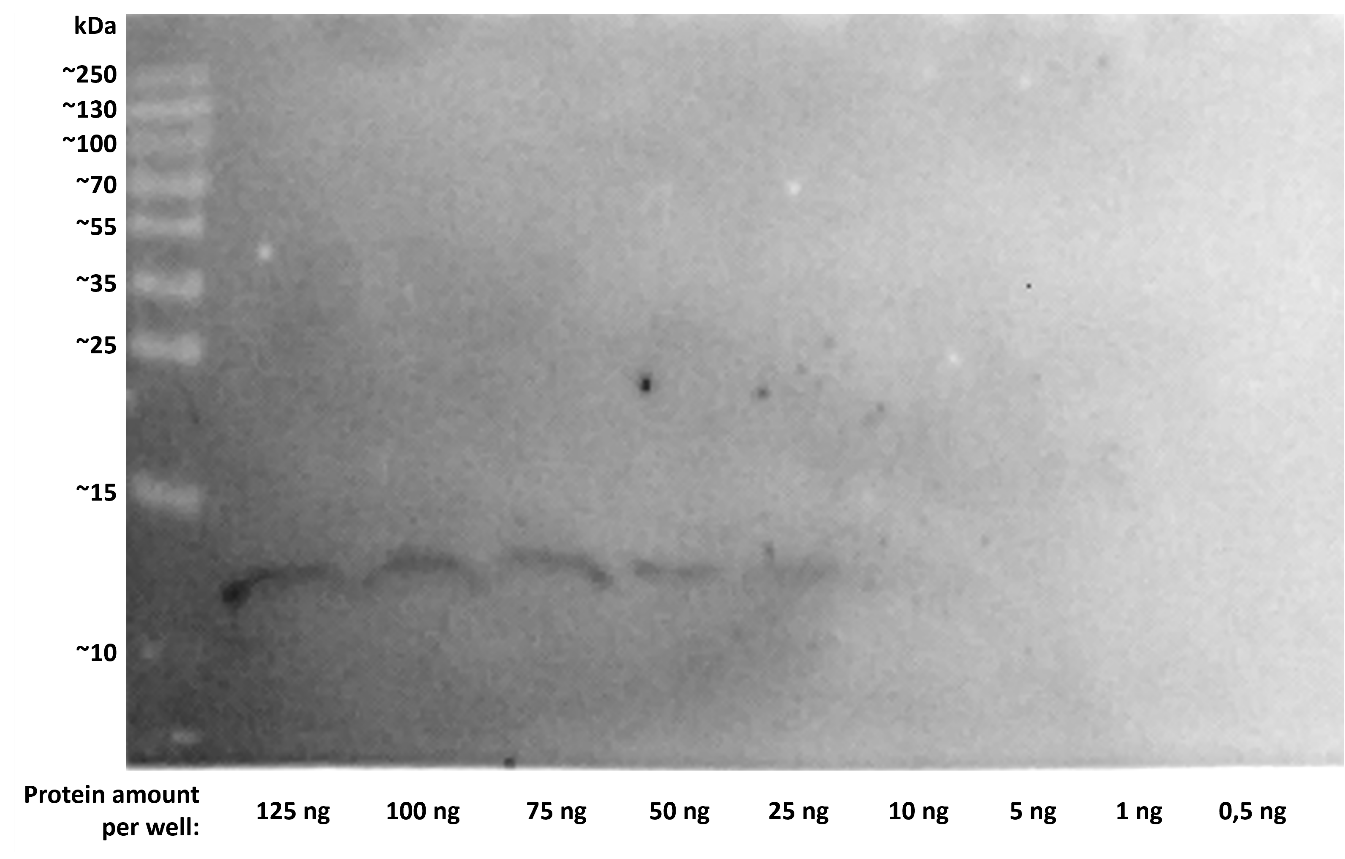
Fig A. Western blot titration of recombinant YqfB protein.** Different amounts of purified recombinant YqfB protein with a 6x-His Tag at the C-terminus were loaded into a gel and the minimal amount that could be detected by Western blot using antibodies against 6x-His Tag (MA1-21315) was established.
