# Supplementary material for: Bacterial amidohydrolases and modified 5-fluorocytidine compounds: Novel enzyme-prodrug pairs
Source: PLoS One. 2023 Nov 30;18(11):e0294696. doi: 10.1371/journal.pone.0294696 (PMC10688628; doi:10.1371/journal.pone.0294696)
Supplement: S3 File — (DOCX) [file pone.0294696.s003.docx]

**Supporting information**

**Proteomic analysis of YqfB and D8_RL**

**Method A: Preparation of cell extracts for proteomic analysis and enrichment of recombinant protein**

Whole-cell extracts of HCT116 and MCF7 cell lines stably expressing YqfB or D8_RL amidohydrolases were prepared by resuspending the cell pellet in Urea lysis buffer (7 M Urea; 2 M Thiourea; 100 mM Dithiothreitol (DTT); 50 mM Tris-HCl, pH 7.4; 1% phenylmethylsulfonyl fluoride, 0.2% aprotinin, 0.2% sodium orthovanadate). The extracts were cleared by centrifugation for 15 min at 12000× g at 4 °C. The protein concentration was estimated using Pierce Coomassie (Bradford) Protein Assay Kit (Thermo Fisher Scientific, Lithuania). Cell extract was enriched for 6×His-tagged proteins 8-fold using HisPur Ni-NTA spin column (Thermo Fisher Scientific, Lithuania). Urea lysis buffer containing 10 mM imidazole was used as equilibration buffer, Urea lysis buffer containing 25 mM imidazole was used as wash buffer and Urea lysis buffer containing 250 mM imidazole was used as elution buffer. Purification procedure was performed according to manufacturer recommendations.

**Method B: Filter Aided Protein Sample Preparation (FASP) for Mass Spectrometry Analysis**

FASP of protein samples was performed using Nanosep®, MWCO 3 kDa ultrafiltration centrifugal units (Pall Corporation, New York, NY, USA) at 13500× g and tryptic digestion were performed according to a modified FASP method described earlier (doi: 10.1021/pr4010019). Cell extract in lysis buffer or enriched fraction in elution buffer were loaded on centrifugal unit to result in up to 200 µg of protein. Sample buffers was exchanged with 200 µl denaturing buffer (8 M urea, 100 mM ammonium bicarbonate (ABC), pH 8.0). Denaturing buffer was exchanged for 100 µL reducing buffer (10 mM DTT in denaturing buffer). The sample in reducing buffer was incubated in a Thermomixer C (Eppendorf, Hamburg, Germany) at 37 °C 600 rpm for 60 min. After incubation, 200 µl of denaturing buffer were added to centrifugal units, after centrifugation, flowthrough was discarded. 100 µl of alkylation buffer (50 mM iodoacetamide in denaturing buffer) were added and centrifugal units were incubated at 37 °C 600 rpm for 60 min. DTT was added to yield a final concentration of 50 mM to deactivate residual iodoacetamide. Alkylation reagents were removed by centrifugation, flowthrough was discarded. Centrifugal units were washed once with 200 µl denaturing buffer, flowthrough was discarded. Three more buffer exchanges were made to FASP digestion buffer (50 mM ABC), 200 µl of FASP digestion buffer were used each time. Proteins were digested overnight in 100 µL FASP digestion buffer with a 1:50 enzyme-to-sample (w/w) ratio using Pierce, trypsin protease MS-grade (Thermo Fisher Scientific, Lithuania). Centrifugal units with digestion reaction mixture were incubated in a 37 °C water bath Sub36 (Grant Instruments, Shepreth, UK) for 16 h. After digestion, the flowthrough was collected into new collection tubes, the centrifugal filter units were rinsed twice with 50 µL FASP digestion buffer, the flowthrough was collected. The solvent from the recovered peptide fraction was evaporated using a vacuum dryer Speed Vac SC110 (Thermo Savant, Waltham, MA, USA). In order to remove volatile salts, dried peptides were resuspended in 100 µl 50% methanol and vacuum dried, procedure was repeated once to remove volatile salts. Dried peptides were redissolved in 35 µl 0.1% formic acid with of 0.2 pmol/µl Hi3 PhosB standard peptide mixture (Waters Corporation, Milford, MA, USA). Prepared samples were analyzed by LC-MS/MS.

**Method C: Liquid Chromatography and Mass Spectrometry**

Liquid chromatography (LC) separation of trypsin cleaved peptides was performed with ACQUITY UPLC I-Class System (Waters Corporation, Milford, MA, USA). Peptides were separated on a reversed-phase analytical column ACQUITY UPLC Peptide BEH C18 Column 300 Å, 1.7 µm, 2.1 mm × 150 mm (Waters Corporation, Milford, MA, USA) at a flow rate of 40 µl/min, 5% solvent B was set until 2.5 min, then a linear gradient from 5% to 35% solvent B was set until 50 min, followed by a linear gradient from 35 to 85% solvent B until 51 min, 85% solvent B until 53 min, linear gradient from 85% to 5% solvent B until 53.5 min, 5% solvent B until 60 min (solvent A: 0.1% formic acid, solvent B: 100% acetonitrile and 0.1% formic acid). The analytical column temperature was set to 40 °C and injection volume was set to 10 µl. The LC was coupled online through an ESI ionization source with Synapt G2 mass spectrometer (Waters Corporation, Milford, MA, USA). Data was acquired using MassLynx version 4.2 software (Waters Corporation, Milford, MA, USA) in positive ion mode. LC-MS data was collected using data-independent acquisition (DIA) mode MSE for 55 minutes. The source/TOF conditions were set as follows: resolution mode, capillary voltage 2.6 kV, sampling cone voltage 40 V, extraction cone voltage 4 V, source temperature 120 °C, desolvation gas flow 800 l/h at 450 °C. During spectral data acquisition, the trap collision energy of the mass spectrometer was ramped from 18 to 35 eV for high-energy scans in MSE mode, for low-energy scans collision energy was disabled. The mass range was set to 50–2000 Da with a scan time set to 0.5 s. A reference compound (2 ng/μl in 50% acetonitrile, 0.1% formic acid) leucine enkephalin (Waters Corporation, Milford, MA, USA) was co-infused continuously at a 8 µl/min flow rate and scanned every 45 s as a reference for accurate mass measurements (reference mass: m/z 556.2771).

**Method D: Data Processing and Protein Identification**

For peptide and protein identification raw data files were processed using ProteinLynx Global SERVER (PLGS) version 3.0.3 (Waters Corporation, Milford, MA, USA). The following parameters were used to generate peak lists: low and elevated energy thresholds, 135 and 20 counts, respectively; reference mass correction window, 0.25 Da at 556.2771 Da/e. Processed data was analyzed using the following parameters: trypsin was selected as a primary digest reagent, one missed cleavage was permitted, carbamidomethylation of cysteines was set as a fixed modification, deamidation of asparagine and glutamine, oxidation of methionine and carbamylation of lysine were set as variable modifications. Minimal identification criteria included 3 fragment ions per peptide, 7 fragment ions per protein and a minimum of 1 peptide per protein. The false discovery rate (FDR) was set to 4%. LC-MS proteomics data were searched against a database of 82,681 protein sequences. The protein sequence database was constructed using sequences obtained from UniProt database (www.uniprot.org): the reference human proteome (UniProt ID: UP000005640), rabbit muscle glycogen phosphorylase (Uniprot ID: P00489) and sequences of recombinant proteins YqfB (10.1038/s41598-020-57664-w) and D8_RL (10.3390/catal10040445).


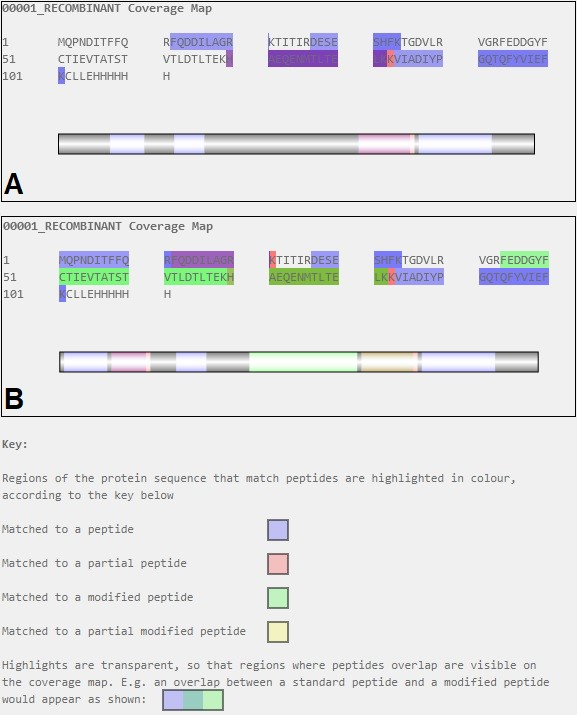
**Fig A. The graphical representation of YqfB sequence coverage obtained after tryptic digestion.** A–YqfB sequence coverage (44 %) result received after replicate “HCT116_YqfB_Ni-NTA_10ul” analysis; B–YqfB sequence coverage (78 %) result received after replicate “HCT116_YqfB_Ni-NTA_10ul_2” analysis. The mass spectrometry proteomics data have been deposited to the ProteomeXchange Consortium via the PRIDE partner repository with the dataset identifier PXD045918. The dataset contains database search result files. In these result files, the YqfB protein has manually assigned “00001_RECOMBINANT” protein.Entry identifier.
