# Supplementary figures and images for: Bacterial amidohydrolases and modified 5-fluorocytidine compounds: Novel enzyme-prodrug pairs
Source: PLoS One. 2023 Nov 30;18(11):e0294696. doi: 10.1371/journal.pone.0294696 (PMC10688628; doi:10.1371/journal.pone.0294696)

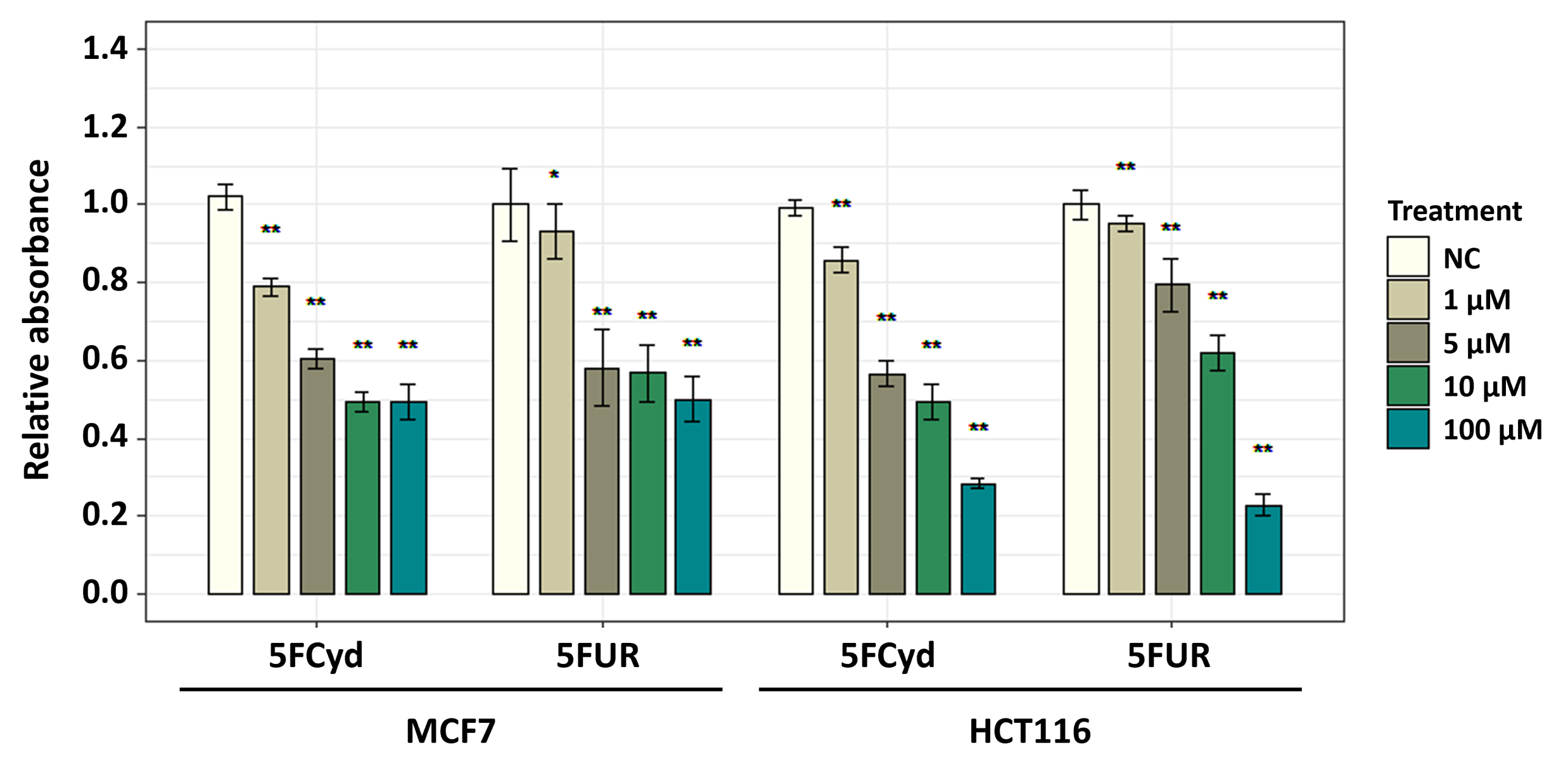

Supplement: S1 Fig — Both cell lines were exposed to concentrations of 1–100 μM of the target compounds for 24 hours. 5FCyd– 5-fluorocytidine, 5FUR– 5-fluorouridine. Statistical significance indicated by p-values, where the symbol * designates p < 0.05, whereas the symbol ** designates p < 0.01 with respect to untreated cells (negative control (NC)). (TIF) [file pone.0294696.s004.tif]
